# Relocating the future: biographical objects, aspiration, and repair in urban Taipei

**DOI:** 10.1111/1467-9655.13863

**Published:** 2022-12-19

**Authors:** Elisa Tamburo

**Affiliations:** ^1^ Harvard University

## Abstract

How might urban relocation unfold as a time of social reproduction? Taking the case of Zhongxin Village in Taipei, a military settlement that was relocated in 2016, I show in this article that while mainland Chinese veterans experienced the move with reluctance, their Taiwanese wives readily stepped up to bridge family histories. Offering new ethnography on ‘family repair’ and the arranging of ancestral altars, I suggest that loss is often not the only force at play when moving home. The Taiwanese wives of Zhongxin Village recount family stories that are elicited through their engagement with ‘biographical objects’. They transmit family lore through daily acts of care even as they project aspirations for the futures of their descendants onto the furnishing of new flats. This marriage of materiality, aspiration, repair, and affect shows that relocation can encourage the social reproduction of the family and, for some, a move from remembrance to aspiration.

## Abstrait

Relocaliser le futur : objets biographiques, aspiration et réparation dans la ville de Taipei

Résumé

Comment la relocalisation urbaine peut‐elle se dérouler comme un moment de reproduction sociale ? En prenant le cas du village de Zhongxing à Taipei, une colonie militaire relocalisée en 2016, l'autrice montre dans cet article que si les vétérans de Chine continentale ont vécu le déménagement avec réticence, leurs épouses taïwanaises se sont volontiers mobilisées pour rapprocher les histoires familiales. En proposant une nouvelle ethnographie sur la « réparation familiale » et l'aménagement des autels ancestraux, l'autrice suggère qu'un déménagement n'implique pas uniquement la perte. Les épouses taïwanaises du village de Zhongxing racontent des histoires familiales suscitées par leur relation avec des « objets biographiques ». Elles transmettent les traditions familiales à travers des soins quotidiens, et dans le même temps, elles projettent leurs aspirations concernant l'avenir de leurs descendants sur l'ameublement de nouveaux appartements. Ce mariage de la matérialité, de l'aspiration, de la réparation et de l'affect montre que la relocalisation peut encourager la reproduction sociale de la famille et, pour certains, le passage du souvenir à l'aspiration.

**Figure 1:**
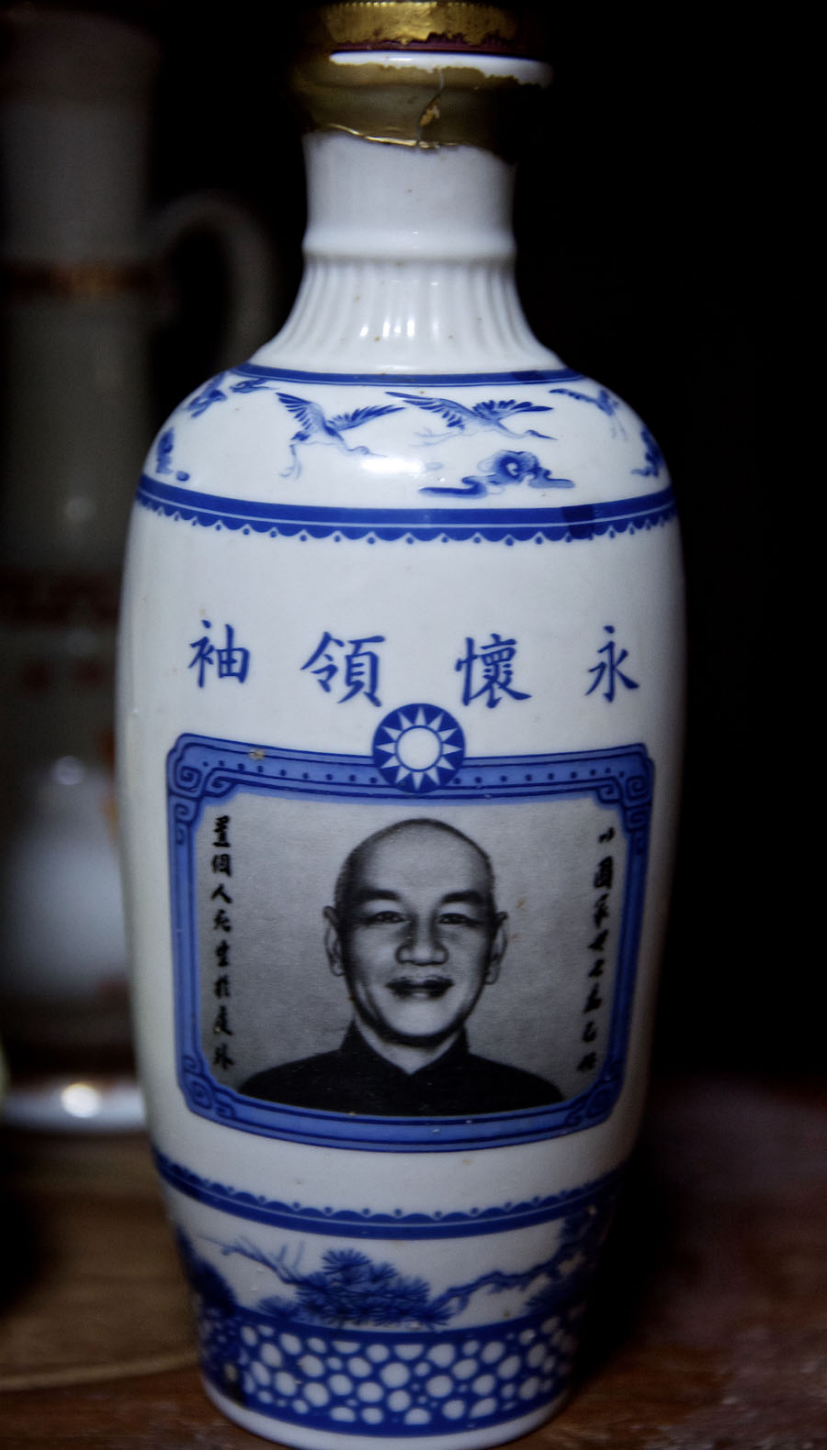
A KMT patriotic rice wine bottle. It reads ‘Forever commemorating our leader (*yonghuai lingxiu*)’. (Photo by the author.)

**Figure 2:**
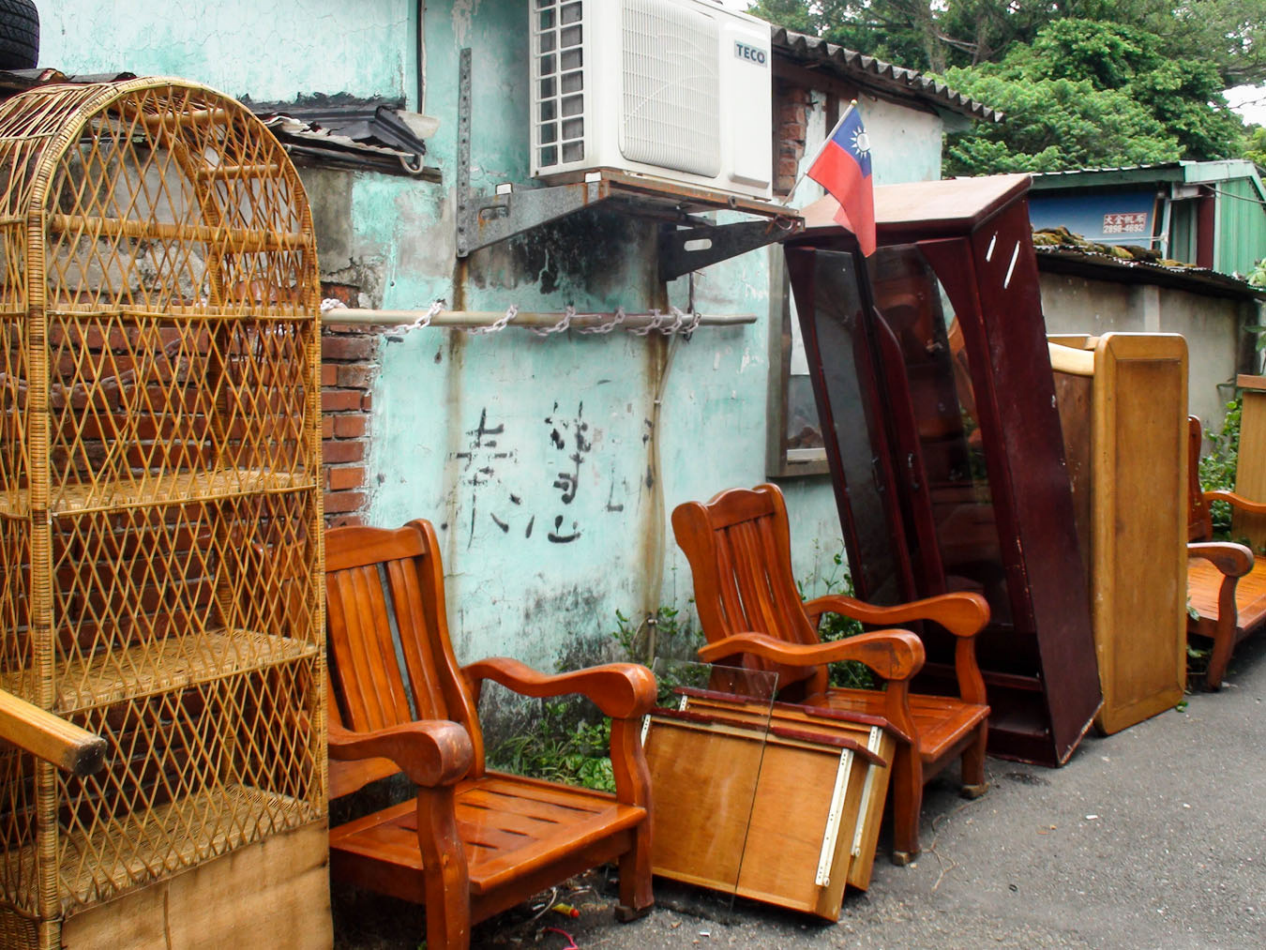
Furniture awaiting to be moved in Zhongxin Village's main alleyway. (Photo by the author.)

On the verge of the move that would see Zhongxin Village relocated to high‐rise buildings, I had spent an entire afternoon with Ms Zheng^1^ tidying up a storage closet located in the loft of her home. Going through the clutter, we found several of her deceased father's possessions, including some patriotic bottles of rice wine (*baijiu*) (Fig. 1). They were gifts he had received when he was a soldier in the army of the Kuomintang (KMT), the Nationalist Party of the Republic of China (ROC). Ms Zheng was a so‐called second‐generation Mainlander, the daughter of a Chinese man who came to Taiwan in 1949 following the Nationalist government's exile from mainland China. With only a few months left before the move, the inhabitants of Zhongxin Village were now intent on clearing their homes and deciding which of their possessions they would bring to their new flats (Fig. 2). At the end of that day of reshuffling things around, packing, and filling up bags with unwanted items, Ms Zheng's mother, Aunt Zheng, settled into sharing memories from her days in the village, including the time when her husband was still alive. In the presence of her daughter, her niece, and myself, she reminisced about when she used to accompany her husband to mainland China to visit his relatives (*tanqin*), something she did until her husband got ill. Recalling the early days of Zhongxin when there were only a handful of houses that eventually became the village, she described her first house in the settlement that was located near to the current main gate and next to a certain Aunt Fan, an old neighbour of hers who at the time owned the breakfast shop that today is still at the village entrance. She recounted stories about her former neighbours, village inhabitants who had moved out before the entire village was due to relocate in 2016.

Clearing their homes prompted the inhabitants to reflect upon their pasts as persons exiled from the Chinese mainland and to renegotiate their future identities and senses of belonging in Taiwan. Zhongxin was a so‐called military dependants’ village (*juancun*), a settlement built to provisionally house the military personnel of the KMT army and their families in the wake of the Nationalists’ exile from the mainland in 1949. In the ranks of the KMT, young men like Aunt Zheng's husband had reached Taiwan's shores with the Nationalist government. Their political expulsion, which was engendered by the Chinese Civil War (1927‐37 and 1945‐49) between the Chinese Communist Party and the KMT, had seen over 1 million people^2^ (Yang 2020: 64) – of which about 600,000 were military personnel – fleeing China with no family or possessions. Although conceived as temporary settlements for military families meant to last only until the Nationalists’ return to the mainland, the KMT failed to win China back and the *juancun* became unlikely permanent homes for many KMT soldiers in Taiwan.

**Figure 3:**
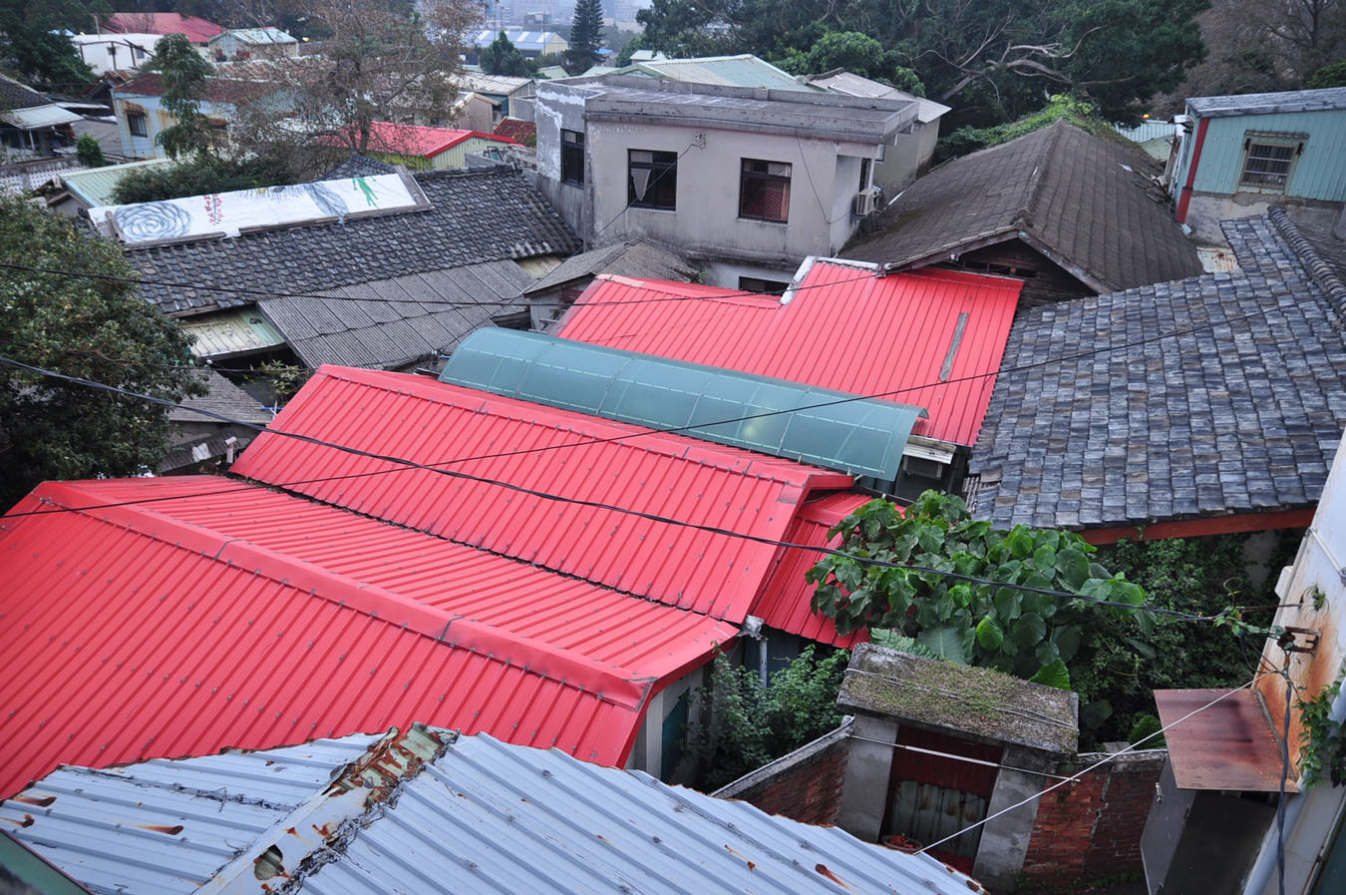
Bird's‐eye view of Zhongxin Village. (Photo by the author.)

Zhongxin Village (Fig. 3) had been built in 1959 in Beitou, a neighbourhood located in northwest Taipei. It had been home to the first generation of mainlander veterans and their Taiwanese wives, their children (also known as second‐generation Mainlanders), and their grandchildren all the way until 2016, when the inhabitants were relocated to high‐rise blocks. For decades, Zhongxin's inhabitants had expected to relocate, particularly at the height of every electoral cycle, when politicians were eager to exchange housing promises for votes. Although in 1996 the Ministry of Defence eventually made provisions to rehouse military families to new flats, Zhongxin had been one of the last villages to relocate when the vast majority of the first generation had reached old age or even had passed away.

By analysing the highly emotive process of clearing old homes and decorating new ones, in this article I show that the relocations of military families unfolded as a time of social reproduction and intergenerational transmission. I discuss the acts of care mobilized through biographical objects during the move, which proved to be an unsettling period of transition, in which affects, identities, and senses of belonging were all threatened by the possibility of loss. By using the term ‘affect’, I mean to call attention to what matters most to people and moves them in life (Lutz 2017: 189), including their personal feelings and emotions, which I understand as ‘relational’ and ‘social’ (Ahmed 2014 [2004]: 8; Lutz 2017: 183; Martin 2013: 157). My ethnography documents the work that emotions do as ‘forms of sociality’ that are mediated by domestic objects (Lutz 2017: 188; Navaro 2017: 211; Navaro‐Yashin 2009: 16).

This understanding of affect does not preclude the possibility that personal emotions may emerge from a charged ‘atmosphere’ (Brennan 2004: 1; Stewart 2017: 192) arising as an inchoate pre‐subjective ‘force’ or ‘intensity’ (Seigworth & Gregg 2010: 1). As I will show, during the months leading up to the relocation, the entire village plunged into deep historical recollection. Preparations for the move reminded the villagers that they would soon leave the homes they had inhabited for over sixty years – a realization that prompted them to re‐evaluate their histories. They furthermore experienced ‘nostalgia’ and ‘reminiscences’ as gendered and generational affective responses to the move. Notably, mainlander veterans approached the transition with resignation, reluctance, and a ‘yearning for what is now unattainable’ (Pickering & Keightley 2006: 920). By contrast, their Taiwanese wives undertook acts of care for their descendants that revealed a treasuring of the past, but also aspirations for a better future, the defying of loss, and preservation of memories about family ancestry. Here, nostalgia and reminiscences performed different social work and enabled distinct engagements with the past. Whereas the veterans’ nostalgia transported them to the reassuring ‘elsewhen’ of the early days of Zhongxin, which gave them a sense of orientation in the unsettled present, their Taiwanese wives reminisced as a way of educating future generations about their family past. Their work of intergenerational transmission emerged even in aspirational terms when decorating new houses and relocating ancestral altars to them further enabled mothers and children to make homes that could ensure the social reproduction of the family.

Stephan Feuchtwang has conceptualized ‘family repair’ as the attempts to maintain family continuity in the face of disruption (2011: 11). For Feuchtwang, family continuity is mostly ensured in rituals, which he suggests enable us to recognize loss, family stories, and even locations that operate as a ‘common reference or place of actual gathering: an old house, a recalled place‐name or simply named and shared places of residence and belonging, tombs and temples’ (2011: 15). Family stories are in particular ‘repeated and shared mementoes, that carry in their retelling passionate concern or on the other hand nostalgia’ (2011: 16). Feuchtwang also calls these moments of storytelling ‘reproductive time’ (2011: 16), a time of repetition that allows family repair to take place.

In Zhongxin, family repair and aspirations for the future of one own's descendants sustain the continuity of both the mainlander patrilineal line of descent and the ‘uterine family’ (M. Wolf 1972: 32‐41): that is, the social unit comprising a mother and her children. With their repair work, the Taiwanese women in Zhongxin Village set out not only to reproduce patriliny (M. Wolf 1972: 32‐41) but also to transmit their husbands’ *mainlander* identity across the generations. These women were key to mediating the first rupture faced by their families in the wake of the exile from the mainland and the subsequent rupture caused by the relocation sixty years later.

Further, by envisaging the new homes in aspirational terms, as dowries and inheritances for their children, mothers strengthen the course of nurturing and care with their children that Charles Stafford calls the ‘cycle of yang’ (2000 *b*: 41‐4). Thus, here patrilineal and uterine families did not oppose one another; rather, the transmission of patriliny and mainlander history occurred *within* the uterine family. Acts of reciprocity among neighbours, known as *laiwang* (Stafford 2000*b*: 38‐9), further contributed to reproducing village solidarity and neighbourly relations in the new setting.

Anthropologists have long examined the relationship between the home, material objects, and persons. Both the material and immaterial geographies of the home are entangled with the meanings and values associated with the domestic material cultures (Blunt & Dowling 2006: 82) that underpin people's ‘appropriation of the larger world’ (Miller 2001: 1). When displacement challenges feelings of belonging, material objects become important statements of identity (hooks 2009: 16; Marcoux 2001: 70; Parkin 1999: 305; Walsh 2006: 138). These objects can embody the self and personhood (Csikszentmihalyi & Rochberg‐Halton 1981: 1‐20; Hoskins 1998: 6), familial and social identities (Chevalier 1995: 27‐8), events (Bryant 2014: 685), kinship ties (Empson 2007: 117), and social relationships (Miller 1998: 5‐7). Having the power to evoke the past (Bryant 2014: 681), objects can furthermore elicit memories, affects, and emotions (Ahmed 2010: 33; Hecht 2001: 123; Navaro‐Yashin 2009: 4). New objects, too, may acquire value and meaning that represent new hopes, aspirations, and possibilities for the future (Clarke 2001: 25), or mediate old and new social relations (Douglas & Isherwood 1996 [1979]: 152; Miller 1987: 5). This is particularly the case for a ‘biographical object’, which Janet Hoskins defines as one that lies on a continuum between gifts and commodities and is enriched with the personal characteristics of the owner. She suggests that biographical objects ‘might be given extraordinary significance by becoming entangled in the events of a person's life and used as a vehicle for a sense of selfhood’ (Hoskins 1998: 6).

On the verge of Zhongxin's move, the ‘biographical objects’ that carried persons’ identities, relationships, and place memories within them called to mind family stories that first generations recounted to their descendants. Some of these biographical objects revealed the uprootedness of the first generation and their exilic condition even as they encapsulated long‐lost kinship ties on the mainland. Mundane items like blankets, tea sets, and perfume bottles reminded the villagers of their long‐lost relatives on the mainland who had gifted them during their family reunions post‐1987 and also bore witness to their own efforts at restoring their family ties through return trips to China. By containing and evoking long‐lost kinship ties, these biographical objects facilitated acts of family repair, *sensu* Feuchtwang.

Revealingly, biographical objects in Zhongxin extended their ‘repair work’ to defy the relocation as a second rupture. While preserving treasured gifts helped the KMT army veterans transfer important aspects of their identity to their new home and reconstitute them there, family ancestral altars restored the family line when their wives relocated them into their new flats. In certain instances, biographical objects led to complex repair work that was filled with contestations and disruptions. Some biographical objects in Zhongxin were alternately discarded, challenged, or dislocated from their owners. These decisions resulted from intergenerational negotiations over the value of the villagers’ relations and possessions, and their practical considerations about their new homes. Yet when ‘repair work’ occurred, it was directed towards asserting identity, transmitting family lore, and ensuring the social reproduction of the family.

Throughout this article, then, I show that just as relocation may unfold like a rite of passage, so persons use biographical objects to manage cultural loss and family disruption. The experiences of Zhongxin villagers, who, by engaging with these objects, alternately enabled or erased their identities, are relevant for reflecting on the wider history and future of Mainlanders in Taiwan. As veterans pass away and the very locations of military villages continue to rapidly disappear from Taiwan's landscape in the wake of its resettlement policies since the 1990s, mainlander culture is at risk of vanishing altogether from the island. While second generations still identify with mainlander culture, their children increasingly identify as Taiwanese (*Taiwanren*). Some openly sympathized with the nativist pan‐green camp^3^ and had supported the Sunflower Student Movement in 2014,^4^ which I witnessed leading to animated discussions between the young and their parents. Ironically, then, it is the veterans’ Taiwanese wives who ultimately transmit mainlander culture to the next generation even as they reshape it through the repair work they achieve with biographical objects.

## Exiled in the city: bamboo fences and mud walls

**Figure 4:**
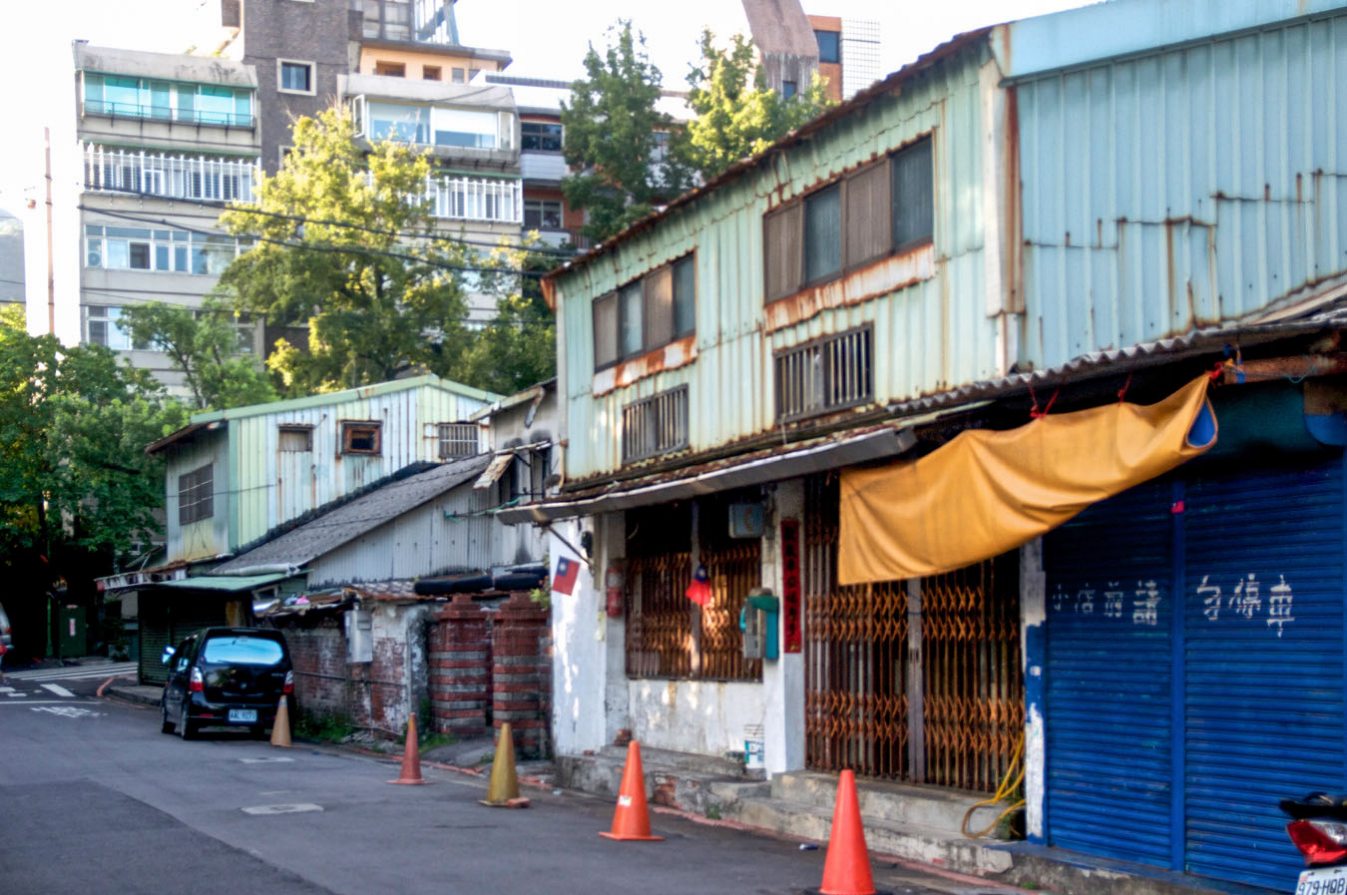
Zhongxin Village's entrance. (Photo by the author.)

**Figure 5:**
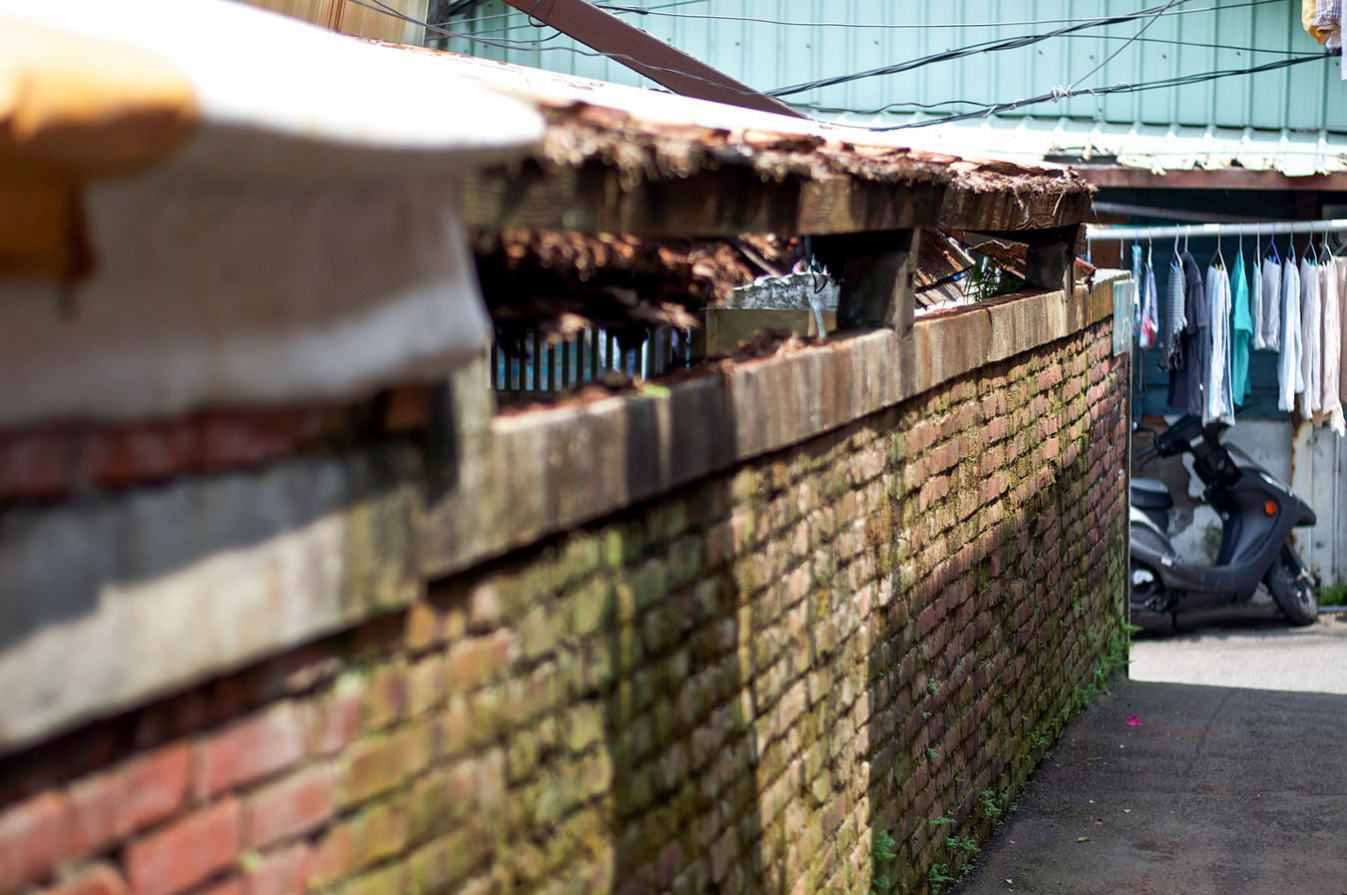
The brick walls of Zhongxin Village's alleyways. (Photo by the author.)

At first glance, Zhongxin Village looked like a concatenation of irregularly shaped shacks at the margins of the main street. On closer inspection, one could notice that each construction had its own character. The two‐storey building at the corner that stretched along the alley was a shop selling breakfast and tea, while a second house separated from the first by the village entrance (*cunzikou*) was built on three levels with a green iron laminate (*tiepi*) roof that lent it a certain sense of impermanence and precariousness (Fig. 4). The transient character of Zhongxin Village emerged from the poor quality of its building materials, exemplified by the mixture of mud and bamboo used to build the house walls. Bamboo was also the material used for making the fences (*zhuliba*) separating one cramped house from another. As anthropologists have observed elsewhere, the flimsiness of bamboo as a building material made it difficult for households to enjoy privacy (cf. Bloch 1995: 78; Daniels 2008: 133; Helliwell 1992: 182). Later, the bamboo fences became brick walls, which formed the little alleys typical of these military settlements (Fig. 5).

The KMT built *juancun* like Zhongxin with makeshift means near military units, including arsenals, military hospitals, headquarters of intelligence services, and military academies to temporarily house the military and their families. ‘Informal military settlements’ (*feilieguan juancun*) also grew on empty land to house the exiled population. In the 1950s, following the retreat to Taiwan, the Nationalist leader Chiang Kai‐shek aimed to reorganize the KMT army and ‘launch a counterattack against mainland China to defeat the communists’ (*fangong dalu*) within five years. It failed, however, and temporary settlements became permanent homes (Fan 2011: 82‐7; Yang 2020: 55).

Zhongxin Village housed mainlander military personnel employed in the adjacent military hospital. While as single men they lived in the hospital dormitories (*sushe*), once married they obtained permission to build their homes on the close‐by empty field. Before the 1960s, the inhabitants of Zhongxin amounted to about 200 households of mixed‐ranking personnel. Higher‐ranking personnel were doctors and head nurses, while lower‐ranking personnel included drivers, technicians, and staff working in the hospital pantry. In the mid‐1960s, some of the hospital facilities moved to central Taipei and higher‐ranking personnel relocated to the city to be closer to their work. The Zhongxin population shrank, even as it accommodated new residents and became a predominantly lower‐ranking village. At the time of my fieldwork, Zhongxin encompassed about eighty households. They included a few mainlander veterans accompanied by their Taiwanese wives, widowed first‐generation men and women, as well as second‐ and third‐generation families, some living with their parents and some by themselves, having inherited the right to live in the settlement after their parents passed away.

After 1949, some of the senior officers already married on the mainland brought their wives to Taiwan. However, young soldiers in Zhongxin Village could not afford the expensive bridewealth demanded by mainlander women in exile. Instead, they wedded Taiwanese women at a lower brideprice relatively late in life.^5^ Many of the veterans’ wives I befriended during my fieldwork were Taiwanese Hoklo,^6^ who were about twenty years younger than their husbands and had been born to peasant families. I met only two senior Chinese women, one of whom passed away during my research, who had themselves experienced the 1949 exile, and a younger ‘mainlander spouse’ (*lupei*)^7^ who came to Taiwan as a marriage migrant much later (Friedman 2015: 7). Afraid of poverty and the bitterness of work in the fields, Taiwanese women married mainlander soldiers, who, despite their low salary, received a fixed income sufficient to support their livelihoods. While Taiwanese women became mediators between their husbands and Taiwan, for instance by helping them communicate in the main local language, Hokkien (*minnanyü*),^8^ they also absorbed mainlander culture and the KMT political affiliation of their husbands.

Military villages like Zhongxin differed from both the Taiwanese urban settlement (*shequ*) and countryside village (*nongcun*) in that residents were not kin. The exile to Taiwan caused major family disruptions and separations (Stafford 2000*a*: 164). Many mainlander soldiers who settled in Taiwan did not return to mainland China until Cross‐Strait travel was resumed in 1987. Some senior soldiers had left their wives and children on the mainland, while young KMT conscripts had abandoned parents and siblings. Those living in military villages were thus bound by the common history of political exile, a shared working unit, and their everyday interactions as neighbours. The crumbling materiality of the crowded houses encouraged close interactions among residents, who actively participated in the lives of their neighbours. People described village sociality as ‘*renao*’, a Mandarin expression which can be translated literally as ‘hot and noisy’ or ‘lively’ (Chau 2008: 485‐6; Steinmüller 2011: 267). In earlier days, women shared food, recipes, and took care of the children collectively, while men worked together in the military hospital. *Juancun* people described the village as a big ‘family’ (*jia*) (Tamburo 2020: 38).

Close inter‐household relationships marked the distinction between insiders and outsiders, which often took the form of ethnic boundaries. Despite mainlander veterans marrying Taiwanese women, these villages became known as Mainlander (*Waishengren*) enclaves, strongly associated with the authoritarian regime of the Nationalist government. The term *Waishengren* – literally ‘people from outside the province’ – is used to identify the mainlander population exiled to Taiwan after 1945‐9, in contrast to the Hoklo (*minnan*) and Hakka (*kejia*) economic migrants who arrived in Taiwan from mainland China pre‐1949. These earlier migrants are called *Benshengren*, ‘people of the province’, often used as a synonym of ‘Taiwanese’.

**Figure 6:**
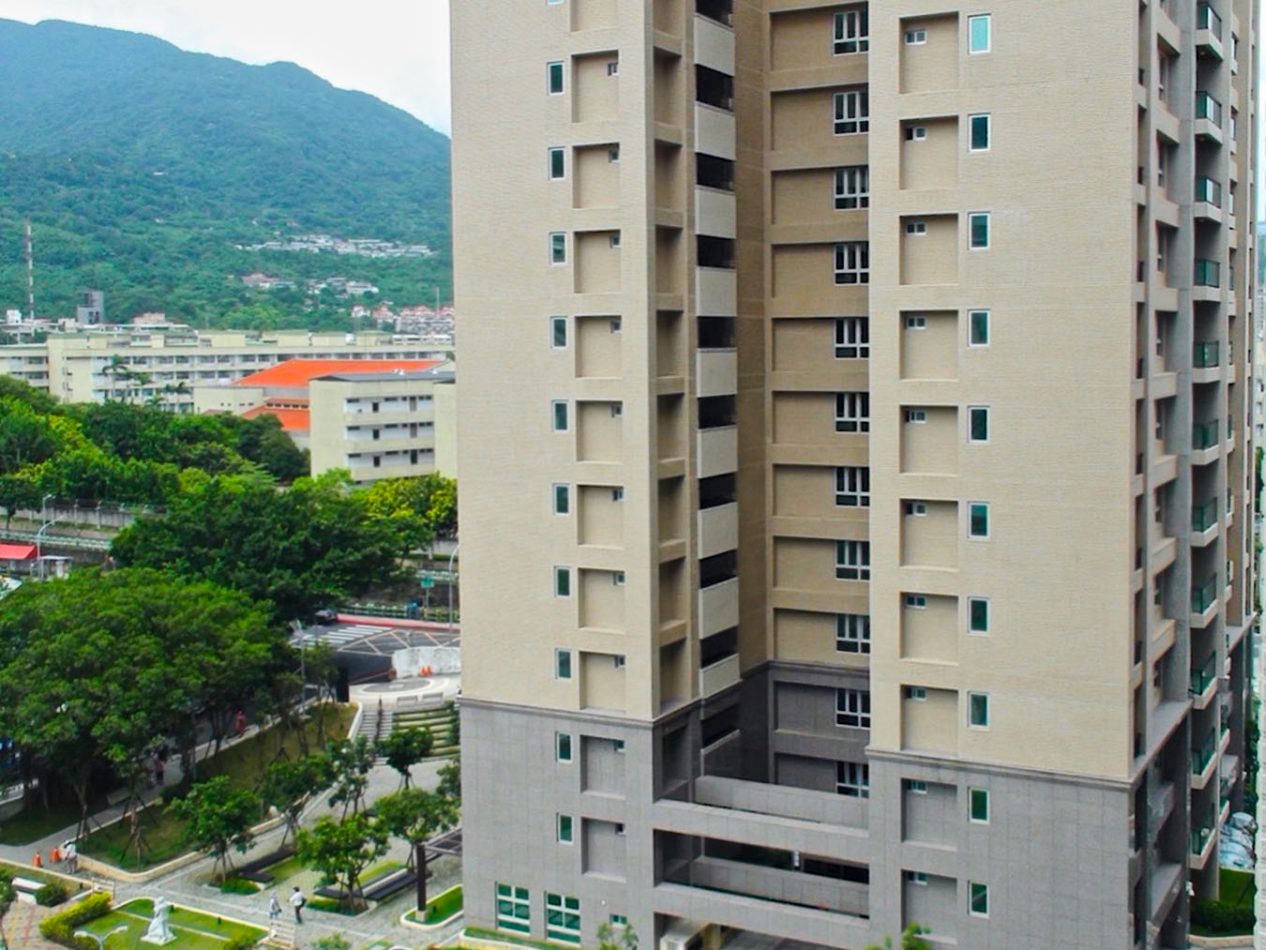
The new high‐rise apartment blocks. (Photo by the author.)

Stepping into this milieu as a foreign researcher meant that I stood out as a stranger, yet through eighteen months of residence in Zhongxin I was accepted as a neighbour of the inhabitants, with whom I eventually became close. It soon became clear to me that mainlander soldiers, animated by a desire to ‘settle down’ (*andun*) after years of ‘wandering’ (*liulang*), had found in the *juancun* a stable home. Despite the poor living conditions and the lack of infrastructure, the villages remained their first home in Taiwan after their exile. Yet, in the 1990s, the state reclaimed public land for urban development. While residents of some ‘informal military settlements’ were evicted without compensation, in 1996 the legislative passed a relocation scheme, the ‘Old Military Dependants’ Villages Relocation Act', which provided the inhabitants of *juancun* such as Zhongxin with subsidized flats in high‐rises. Officially designed to offer stable housing to military families, the scheme also aimed to ramp up urban development in Taiwanese cities. After decades of waiting, Zhongxin had been one of the last military villages to relocate to a block of four high‐rise buildings in October 2016 (Fig. 6). Together with the inhabitants of three other small military villages, Zhongxin's residents would be distributed across different buildings. While the veterans had long aspired to higher‐quality housing, now reaching an elderly age, they were reluctant to be displaced again. Nevertheless, as the inhabitants prepared to move, their recollections and aspirations for the future filled the air.

## Relocation as loss: materiality and displacement

**Figure 7:**
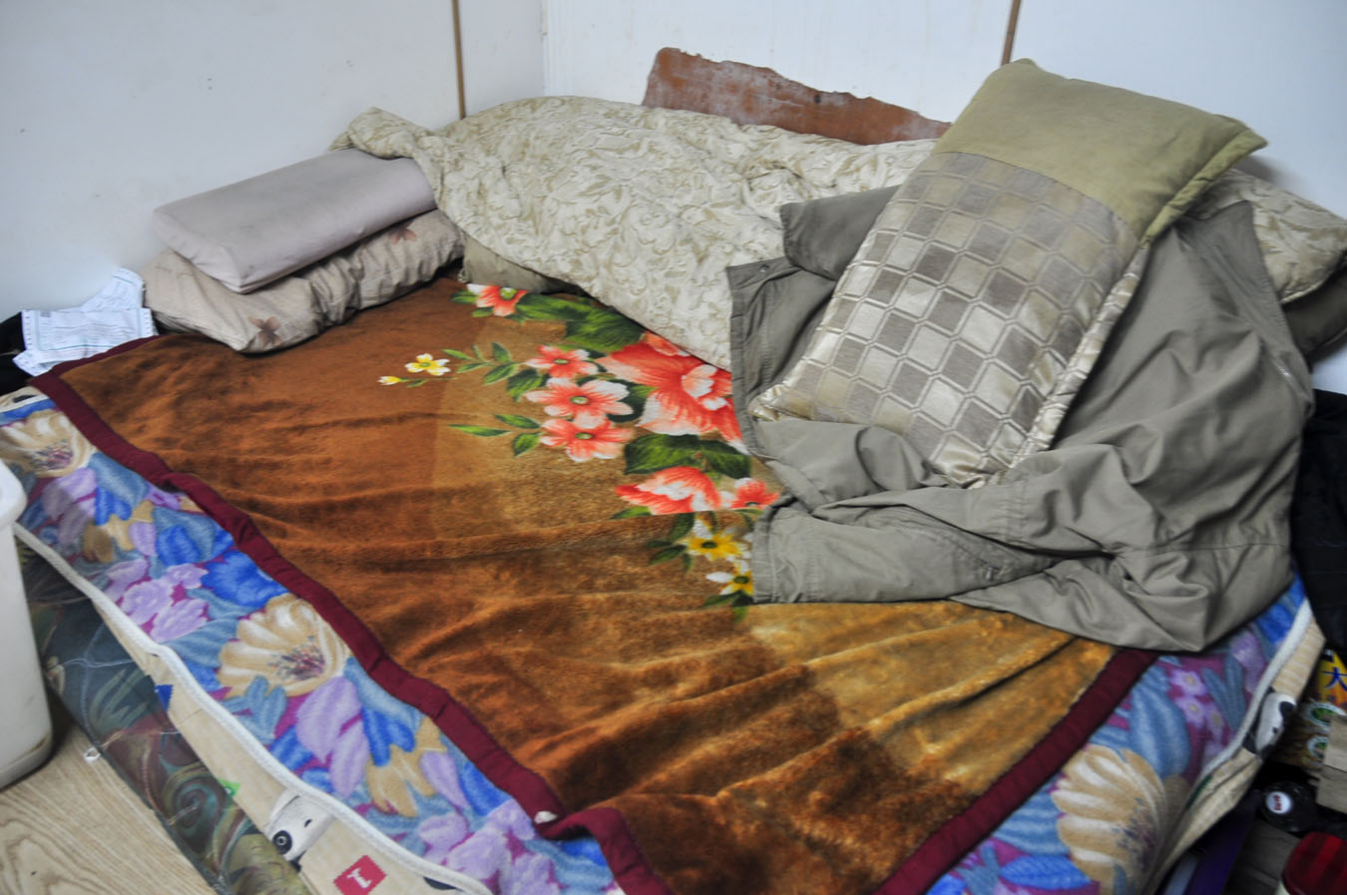
Uncle Wang's gifted blankets. (Photo by the author.)

**Figure 8:**
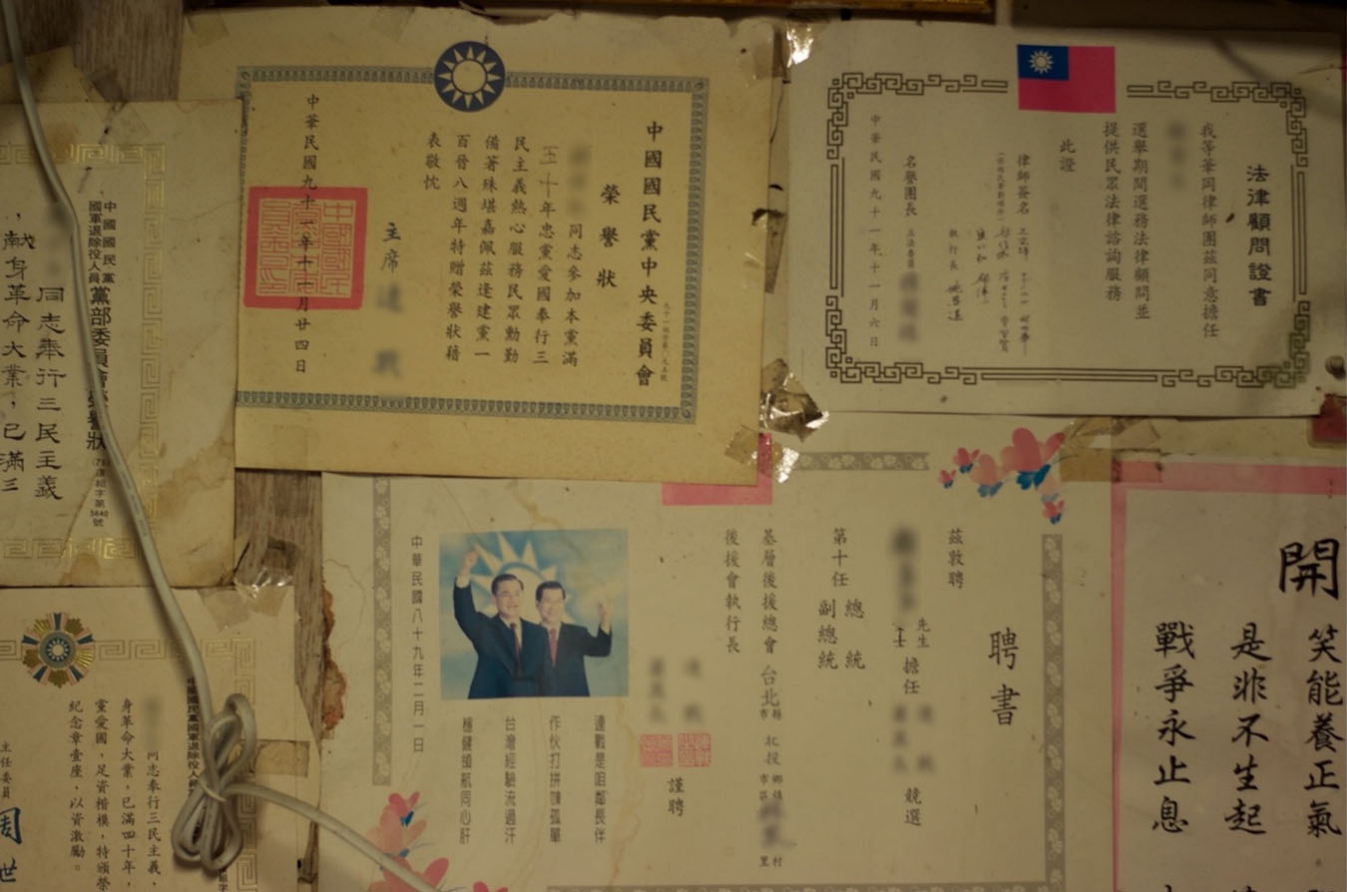
Uncle Wang's military awards. (Photo by the author.)

The widowed 91‐year‐old Uncle Wang was saddened at the idea of moving. He told me that he would stay until the end. Ever since his arrival in Taiwan in 1949, he had been living in the three‐storey house next to the village entrance. As residents prepared to move, I visited Uncle Wang once more to enquire about his arrangements for the relocation. ‘Do you already know what you will bring with you?’, I asked. He first indicated some blankets scattered on his bed, adding that they were a gift from some of his relatives in mainland China, which made them a treasured possession (Fig. 7). They had been given to him on one of his trips back to the mainland after Cross‐Strait travel had resumed in 1987. For Uncle Wang, who had been living in exile his whole life, the blankets were a memento of kinship ties on the mainland and a reminder of his origins. When I asked what else he would bring, he indicated the many military awards covering the walls of his living room (Fig. 8). Like many others in Zhongxin, he was a low‐ranking soldier in search of recognition. These framed awards, which hung all over the room, had been the centre of my first conversation with him when we met about sixteen months earlier. Narrating his war stories in which he used to describe himself as a hero (*yingxiong*), he had called me inside his house to proudly show his awards. These honours demonstrated the key role he had played in the ranks of the KMT. It became apparent after only a few visits that Uncle Wang was very attached to his awards, which were not only mementoes of his experience of war and exile as a KMT veteran, but also an integral part of his identity.

Veterans like Uncle Wang were reluctant to leave. Like their wives, veterans had aspired to a better house for decades. Yet these were being allocated when they had now reached old age, thus leaving them disoriented about how to start a new life at the end of their lives. They believed that while the new houses were officially conceived for their generation, they were in fact destined for their children and grandchildren. But even as they sought to escape an unsettling present through their nostalgic associations to a comforting past – which notably did not equate with material comforts – they had to prepare to leave, aided by their children, grandchildren, and daughters‐in‐law. Faced with the possibility of loss, they negotiated their identities through biographical objects in the home that became the containers through which they transferred important aspects of their identity to their new flats.

Uncle Wang's awards revealed his identity as an exiled KMT veteran. With his collection of medals, prizes, and certificates, he could impress curious passers‐by who, intrigued by the unusual architecture of the settlement, would visit his house and listen to his numerous heroic war accounts. Prizes created his authority, his status, and his popularity inside and outside the village. The awards cued visitors to stop by for a chat, which Uncle Wang relished and which made his day worthwhile. Thus, as biographical objects, his awards mediated his identity and social relations.

**Figure 9:**
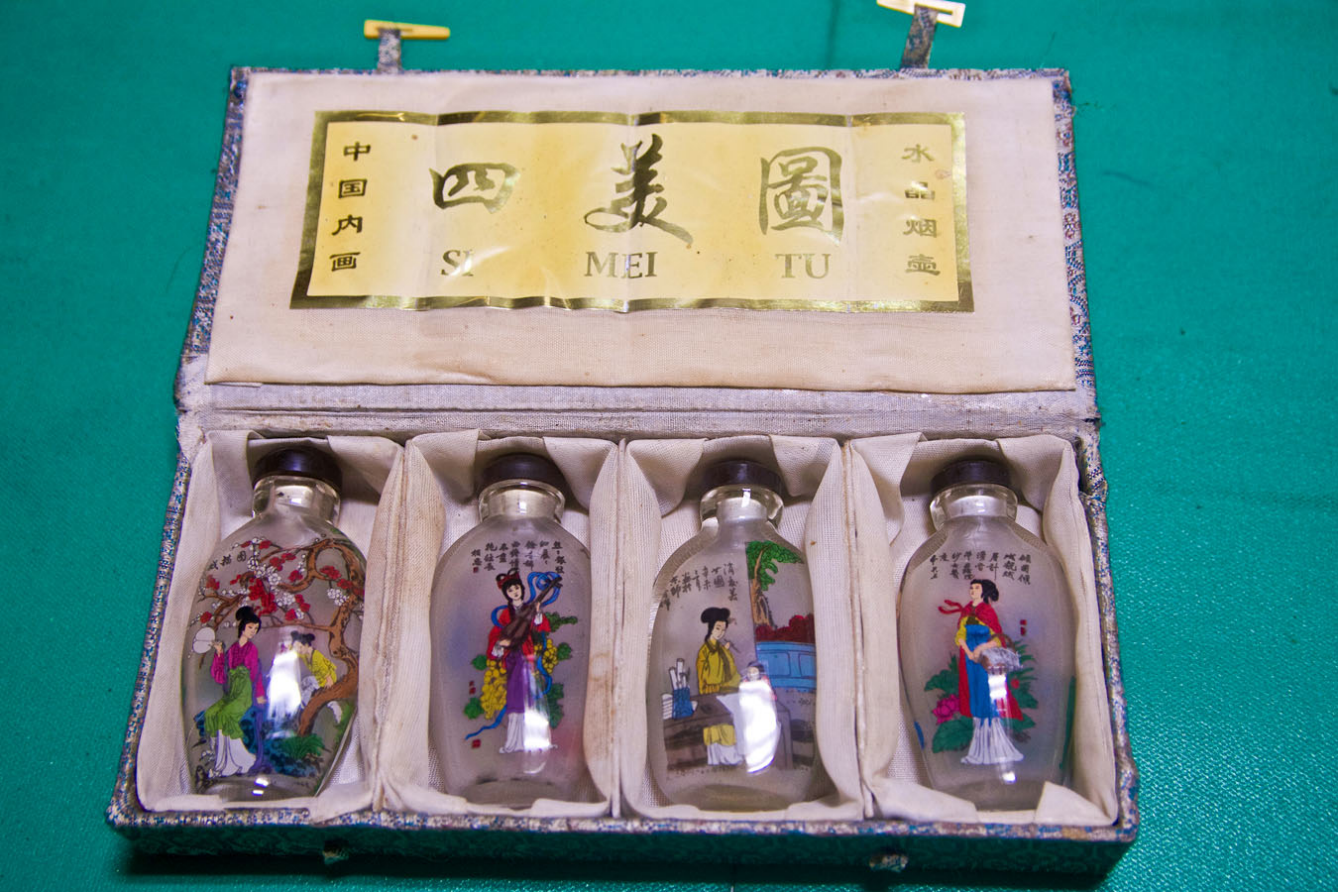
Uncle Sun's gifted snuff bottles. (*Photo by the author*.)

Some of Uncle Wang's other biographical objects, such as his blankets, revealed the uprootedness of the first generation. In 1949, mainlander refugees landed in Taiwan with no family and virtually no possessions. When veterans could return for the first time to the Chinese mainland in 1987, they found much had changed in their places of origin: their parents had passed away, their natal homes had been demolished, and their towns had urbanized to the extent of becoming unrecognizable. They, however, had also reunited with some of their close family members, like their siblings, and had reacquainted themselves with more distant relatives such as cousins, uncles, and aunts. These post‐1987 trips to mainland China allowed ‘family repair’ work to take place in the wake of political exile. Other neighbours in Zhongxin Village had described these reunions with long‐lost relatives as important occasions to reconstruct family bonds. Often these gatherings involved shared meals and gift exchanges. Aunt Li showed me, for instance, some tea sets that her husband's relatives had gifted her family on one of their trips to Tianjin, her husband's natal city. Similarly, Uncle Sun showcased two sets of small painted glass perfume bottles, once also used in China as snuff bottles (*yanhu*), which had been gifted to him by his sister during a trip back to Shandong province (Fig. 9). When physically distant from kin, domestic biographical objects like these can reflect, and even augment, social relations by separating and containing parts of a person's biography that are temporarily located elsewhere (Empson 2007: 114). By evoking separations and reunions with long‐lost relatives stretching over decades, biographical objects, moreover, built temporal bridges between people and their own kin. Against the background of Uncle Wang's exilic condition, biographical objects became spatio‐temporal reminders of his kinship networks on the mainland, thus performing ‘family repair’ in the aftermath of his exile.

In the context of the move out of Zhongxin Village, biographical objects became even more important as they transitioned the inhabitants’ identities and stories as displaced Mainlanders to a new housing complex that exhibited little or no material reference to the history of their exilic condition. David Parkin discusses the relationship between identities and possessions during displacement in Africa, pointing out that forced resettlement opens a space of vulnerability that is ‘as existential as it is material’. These two aspects of vulnerability are entangled ‘for it is through the skills and objects one may take that one's future may be given shape’ (Parkin 1999: 305). Non‐commodity, gift‐like objects are inscribed with stories, dreams, the transmission of skills and status, and may temporarily encapsulate personhood. As ‘transitional objects’ carried by people on the verge of forcible human displacements, they are ‘mementoes of sentiment’, which not only illustrate their identities in flight but also offer them the possibility of re‐personalizing their homes in the aftermath of resettlement (Parkin 1999: 303). Relocations like these present inhabitants with the possibility of losing their sense of self, social identity, and relationships, but also bring them into close contact with possessions that evoke life stories and memories of the past. People are thus confronted with the ‘problem of how to convey the stories and emblems of self and culture’, a problem which becomes clear at the point of departing from a place, when choices need to be made about ‘what to take and what to leave behind’ (Parkin 1999: 308).

Seen in this light, when faced with the potential losses to be incurred through relocation and the consequent separation of neighbours across different buildings, biographical objects become important assertions of identity. In Zhongxin, those biographical objects which once enabled family repair regarding veterans’ kinship ties on mainland China in the exile's wake now stave off the displacement of personal and familial identities characterized by an exilic condition vis‐à‐vis the relocation and its social reconfiguration (cf. Tamburo 2020). Through these objects, repair work in Zhongxin extends from mending long‐lost kin and ancestral relations to reconstituting oneself as a mainlander person outside of the historical, diasporic, makeshift settlements in Taipei and its neighbourly environs.

**Figure 10:**
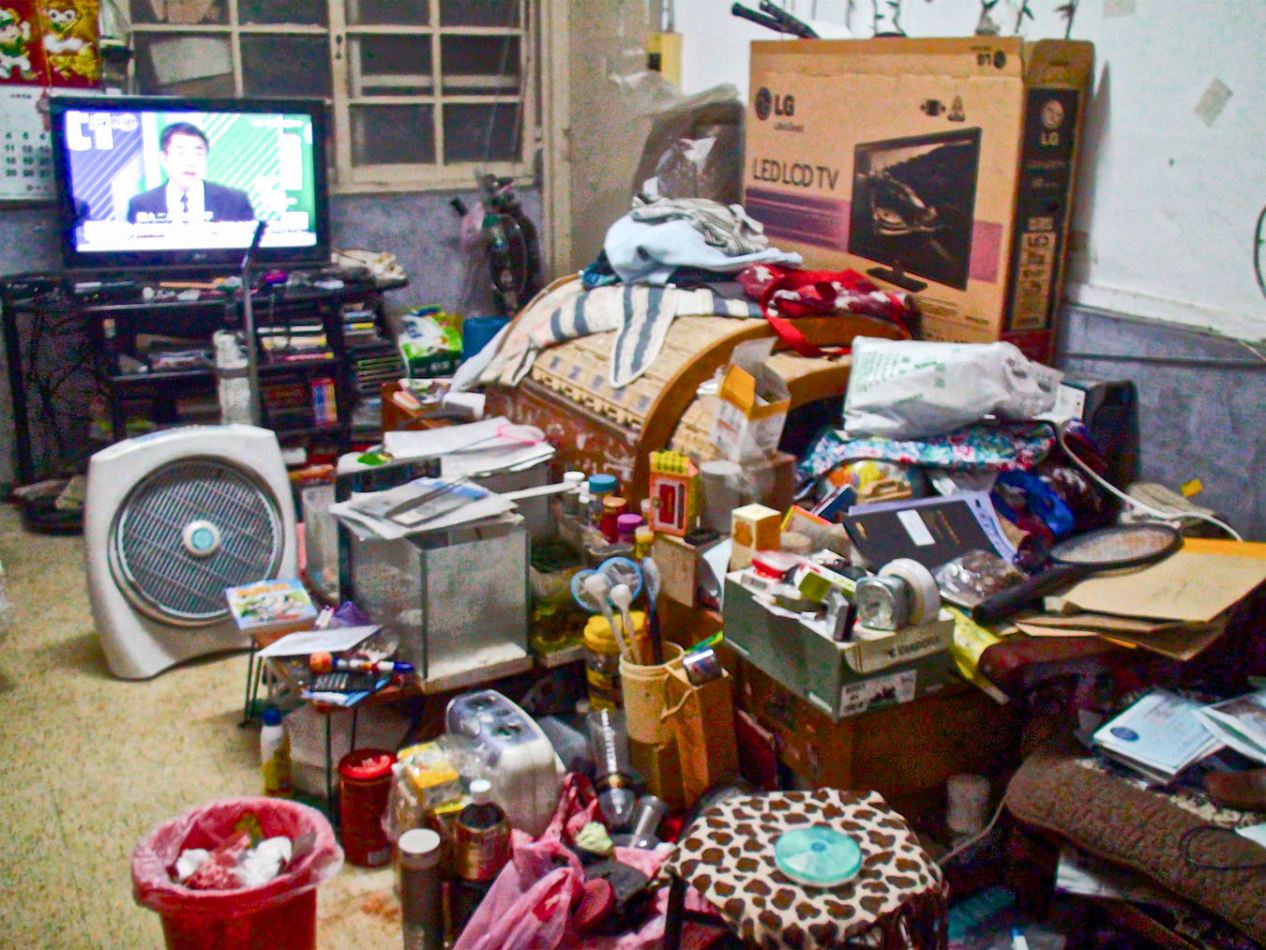
Clearing homes in Zhongxin Village. (Photo by the author.)

Biographical objects thus help villagers to place the ruptures and losses of multiple relocations into a wider trajectory that encompasses their experience of double displacement in Taiwan. As Rebecca Bryant suggests, these objects mediate claims to belong to a place, a community, and to history (2014: 683). Let us now return to the opening ethnographic vignette to this article and consider how the veterans’ wives reminisced about the past and managed their household possessions during the move to repair the rupture of relocation (Fig. 10). Here, Taiwanese women perform the essential duties of care in preparation for departures, as they are the ones entrusted with the continuity of society.

## Clearing old homes: reminiscing about the past for intergenerational transmission

Three generations of female family members gathered in Aunt Zheng's living room after an afternoon of home clearing, where ‘*Ahma*’ regaled her daughter, grandchild, and me with her story. She reminisced about past village life, her old neighbours, and her trips to mainland China to visit her husband's relatives, all of which conveyed the mainlander ancestry of her family, the exilic condition of her husband, and the attempts to mend long‐lost family bonds. During fieldwork, I became a close acquaintance of Aunt Zheng and knew her as a Hoklo woman who, although born and raised in Taipei, strongly identified with her Fujianese ancestral origins in mainland China, as well as with her husband's mainlander family line in Hunan province. On various occasions, Aunt Zheng expressed her pride in her family's mainland Chinese ancestry and that she wanted her grandchildren to know where their family had come from.

Aunt Zheng used engaging storytelling as a caring act of both intergenerational transmission and family repair. Like many other widowed Taiwanese women, she stepped in to transmit the family lore and mainlander patrilineal ancestry in the absence of her husband, when many veterans like him were disappearing along with their mainland culture. Acts of family repair may especially occur in contexts where ‘younger generations have turned their back to their grandparents’ past’ (Feuchtwang 2011: 11). The end of the KMT authoritarian period ushered in by the lifting of martial law meant that Taiwanese people could redeem their rights, identity, language, and history after years of political repression. Following democratization, Taiwanese nationalism and its independence movement surged, and while the so‐called ‘Mainlanders of the third generation’ increasingly identified as Taiwanese, the first and second generation felt out of place (Li 2015 [2002]: 119; Yang 2020: 218‐28; Yang & Chang 2010: 109). It is in this context that women like Aunt Zheng took to heart the education of their grandchildren about their Chinese ancestry and the troubled historical conjunctures that brought their grandparents to Taiwan.

**Figure 11:**
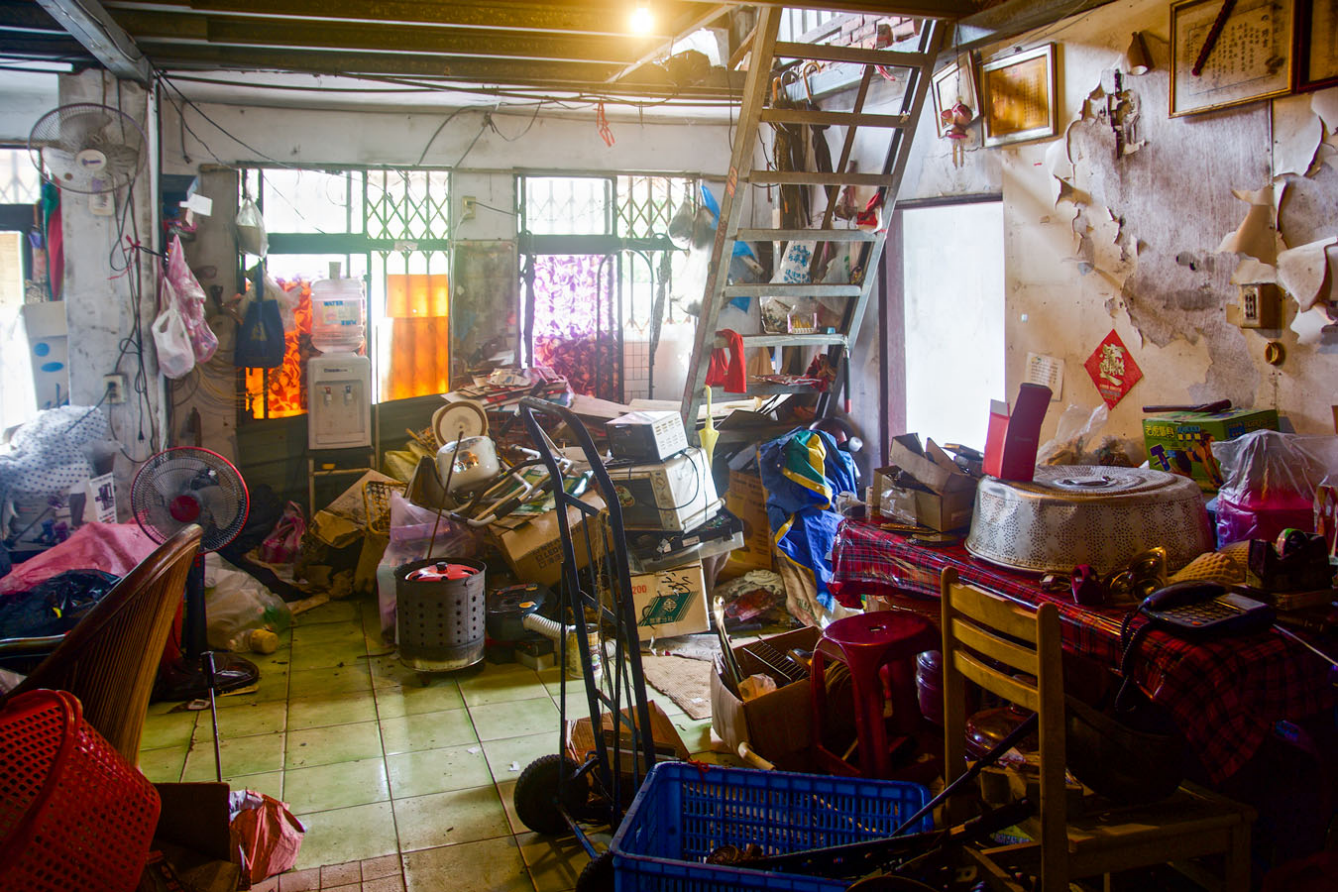
Unwanted items in Zhongxin Village. (Photo by the author.)

While family repair unfolded through engaging storytelling, it extended to the task of managing unwanted items in the house. But choosing what to preserve, donate, sell, or get rid of implied valuating items along with the appraisal of one's own social relationships. Therefore, the acts of family repair undertaken by Aunt Zheng and other Taiwanese women were not devoid of tensions. Persons from different generations may make different choices about the value of specific objects that may even call into question whether they count as biographical objects in the first place. Some of their decisions may lead to reproducing social relationships outside the agnatic group that favour Taiwanese women's families of origin or neighbours. This happened in Aunt Zheng's case and, as her daughter predicted when officials announced the move for Zhongxin Village, it even led to conflicts between her and her mother. The conflicts were sourced to her reluctance to throw away anything. Tensions came to a head when Ms Zheng gave some clothes to Uncle Wang's son, Little Three, who had helped the neighbours clear their homes of unwanted items. He carried things away on a heavy‐duty trolley to his living room, which had temporarily become a depot for things to be sorted and then thrown away or sold (Fig. 11). Uncle Wang worked with Little Three to support his family of five, making some extra cash on top of his veteran pension. Some villagers purposefully donated items to Uncle Wang to help him and Little Three make extra money, evidencing the ‘cycle of *laiwang*’ (Stafford 2000*b*: 38‐9), reciprocal support and care among neighbours, that was the backbone of their village sociality.

After a busy day of house clearing, Ms Zheng was helping Little Three carry away the trolley full of clothes she had prepared for him when her own mother – Aunt Zheng – suddenly stumbled out of the house in anger. Watching the scene with a betrayed look on her face, Aunt Zheng immediately went back inside. Only later that day did Ms Zheng explain to me that among the clothes there were brand‐new ones, all bought by Aunt Zheng for her grandchildren, which had been forgotten in the wardrobe. Her grandchildren had long outgrown them. While Aunt Zheng wanted to give the clothes to her sister's children with kids so that they could wear them, Ms Zheng considered this to be ‘burdensome’ (*ma* *fan*), as she had once stored something for a relative who forgot to pick it up, and it had stayed in her house for months before she eventually threw it away. Still, Aunt Zheng could not accept that her daughter had so little consideration for her own kin that she would prefer donating things to Uncle Wang to be sold rather than giving them to her own matrilineal relatives. For Aunt Zheng, giving away things to strangers was different to distributing items within her kinship group: the fact that these were ‘your people’ and not ‘some stranger out there’ made all the difference.

‘Elderly people come from a time when they had nothing, and now they want to keep things if they still work’, said Ms Zheng. ‘Take clothes, for example, they think that it is still possible for their children to wear them. But we will not because those clothes belong to another time, another epoch, a distinct sense of fashion. We don't want them’. Objects can thus hold distinct regimes of value and meaning for different generations (Kopytoff 1986: 80): for Aunt Zheng, who lived through years of scarcity, clothes are important possessions which, if unused, could be gifted to matrilineal kin; Ms Zheng, though, cared about preserving other items. She taught her children to hold onto objects that might one day elicit fond memories, such as school notebooks that they one day should show to their own children. Whereas Aunt Zheng sought, as a widow, to repair her husband's lack of family ties by redistributing objects to members of her family of origin outside the village, her daughter – who had grown up in the absence of close agnatic relatives – prioritized the neighbourly relationships at the heart of village solidarity which had informed her biography. She donated the excess clothes to Uncle Wang to support his livelihood, knowing that he could make a little extra money from the second‐hand sale. In each case, redistributing biographical objects involved an intergenerational valuation of one's own possessions and social relations.

Storytelling and redistributing biographical objects were, however, not the only strategies that Taiwanese women followed to reproduce the family. Mothers and children refurbished and decorated their newly allocated homes together in ways that expressed their aspirations for the future while reinforcing the bonds of the ‘uterine family’. Concerns over dowry and the inheritance of new assets came into focus here, as women sought to produce new selves and obtain a higher social status.

## Decorating new homes: aspirations for the future

Families did not simply decorate new flats by buying new furniture but also engaged in larger‐scale renovation. Changes included demolishing internal walls to change the layout within flats: for example, by opening the kitchen to integrate it with the living room or creating larger bedrooms. Better‐off families invested in plaster decorations, false ceilings, and ceiling lights for their living rooms as well as in changing the tile flooring into parquet. Fewer households changed bathroom tiles and fixtures. In refurbishing their new flats, Taiwanese wives revealed their aspirations for the future of their offspring, their family status, and the refashioning of selves. Refurbishing work expressed the aspirations, creativity, and tastes of the second generation, who had their mothers’ approval.

A designer by profession, Hannah spent most of her free time planning and designing the interior of her new flat. Like most second‐generation inhabitants, she cherished her childhood memories in Zhongxin but believed that the village had become unliveable. She wanted to expand her new home's living room by turning a standard bedroom into a Japanese‐style bedroom with a raised floor covered in tatami and sliding doors opening onto a more spacious living room. Hannah's mother, Aunt Xu, was happy to leave her daughter in charge of the decorating process: ‘She is a professional designer, so she knows what she is doing’, she said. ‘One day, the house will be hers, so it is good that she designs herself’. By renovating, Aunt Xu invested in an asset which, she hoped, would provide a good dowry for her daughter and bring her a good marriage. Daniel Miller argues that it is through the medium of selected goods that ‘shoppers develop and imagine those social relationships which they most care about’ (1998: 5). Similarly, the veterans’ wives in Zhongxin Village furnished and decorated their new houses with the dowries, inheritances, and futures of their descendants in mind. Renovating their new homes served here to strengthen nurturing relationships between mothers and children.

On another level, practices of home decoration involved the refashioning of selves. The third generation sought privacy, comfort, and a personalization of their living space that matched their tastes. The Wu sisters, for instance, surfed the internet, chose new wardrobes, and worked to combine the designs and colours they liked most. Together we went to see new furniture in a DIY store, where they bought wall paint to personalize the colours of their bedroom walls. They looked forward to inhabiting the new homes and aspired to having private bedrooms and a higher quality of living. These desires call to mind the pursuit of private, middle‐class life in the 1990s by many young mainland Chinese in response to the opening reforms and the subsequent rise of individualism in China (Kleinmann *et al.* 2011: 15‐18; Tomba 2014: 29‐61; Yan 2003: 112‐39; 2009: 1‐24; 2011; 41‐7; Zhang 2010: 107‐36; Zhang & Ong 2008: 15‐19). Although the meaning of these aspirations needs to be weighed against China's socialist past of work units (*danwei*) and collectives, something far from Taiwan's experience, the flimsy mud walls of the crowded buildings in the military settlements of Zhongxin equally offered little privacy. The younger generations were thus eager to enjoy the comforts of new homes – even if this would come at the cost of neighbourly solidarity and comradeship that had been the backbone of Zhongxin's sociality (Tamburo 2020: 38‐41).

Decorating was also a new marker of class and desires for social distinction. Previously buried under the disrepair of old village houses, military ranking had not been reflected in the old housing arrangements. But gaining a subsidized new flat enabled villagers to create novel forms of social distinction à la Bourdieu (2002 [1977]: 82‐3; 2010 [1979]: 192‐7). Not every family could afford to spend a fortune on decorating. Many took up a mortgage to pay the outstanding 20 per cent on their flat, but this did not prevent less well‐off households from making home improvements. Uncle Wang's daughter added newly made gold cushions to the old wooden couch and chairs in her rearranged living room that renewed it with a fresh look, even giving me the impression that the chairs had been replaced with new ones.

The purchasing of new ancestral altars made family distinctions particularly visible. Better‐off families spent several thousand NTD (New Taiwan dollars) to buy new ancestral tables, tablet cases, and ritual objects such as lamps. Notably, Taiwanese wives projected many of their aspirations onto family ancestral altars, which they refashioned as they newly purchased wooden tables and ritual objects. They strived to provide an adequate residence for the ancestors that would not only meet the modern standards of new flats but also encourage them to support the younger generations and their own futures. Through their creative approaches to moving and refurbishing altars in new flats, Taiwanese wives preserved the tradition of ancestral worship, transmitted patriliny, and commemorated mainlander family history. Women thus managed family relationships to the ancestors in ways that bridged their recollections of the past and their aspirations for their descendants.

## Synthesizing past and future: a new home for the ancestors

In Greater China, the cult of ancestral worship (Ahern 1973: 91‐149; A.P. Wolf 1974) strongly relates family history to locality (Feuchtwang 2001: 22; Jing 2003: 115; Stafford 2003: 14‐17), but for Taiwan's Mainlanders it is also a potent reminder of separation from their places of origin on the mainland. Since Han Chinese on the mainland keep their ancestral tablets in a shrine within the house of origin, most Mainlanders had no choice but to leave them behind after their exile to Taiwan. Exiles thus lacked the material manifestations of both their ancestors and their family history in their domestic space. Some of my interlocutors told me that they had resorted to writing their ancestors’ names by hand on the backs of new wooden tablets bought in Taiwan. Others said that they did not discover their parents’ birthplaces until they checked the original tablets located in mainland China after Cross‐Strait travel resumed in 1987.

**Figure 12:**
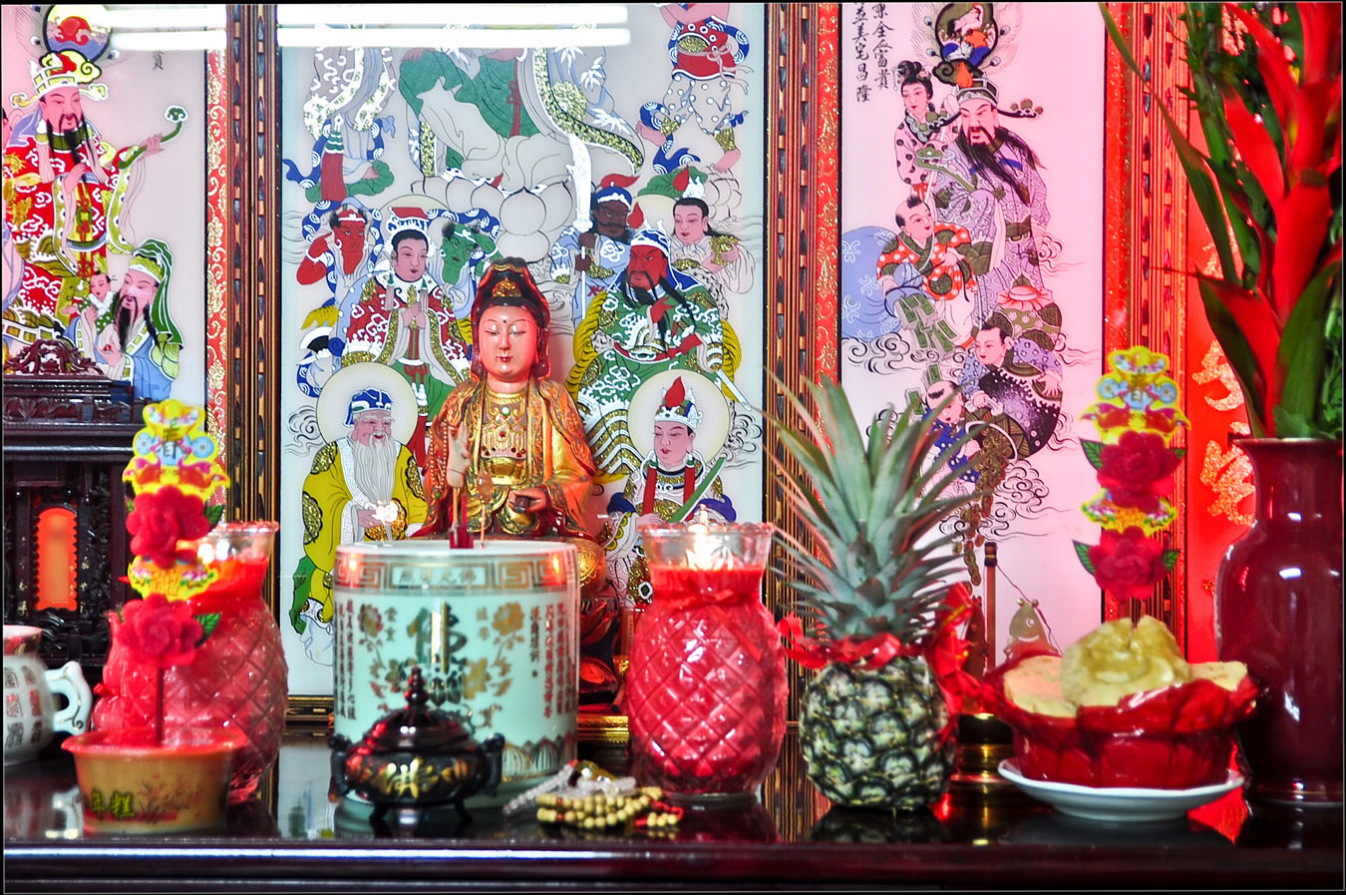
A Taiwanese ancestral altar in a Zhongxin's home. (Photo by the author.)

Revealingly, in Taiwan, the ancestral tablets of mainlander families take Taiwanese material cultural forms. The right side of a Taiwanese ancestral altar is reserved for the gods, where the statues or imagery of deities such as Guanyin or Tudigong^9^ are placed, while the ancestral tablets are kept in a wooden case on the left (Fig. 12). In Zhongxin Village, each household found a different way to relocate their ancestors and preserve their family history. Some residents brought the ancestors to their new homes, purchasing nothing new, while others bought new tables, cases, and accessories that they felt would be worthy of their modern flats.

**Figure 13:**
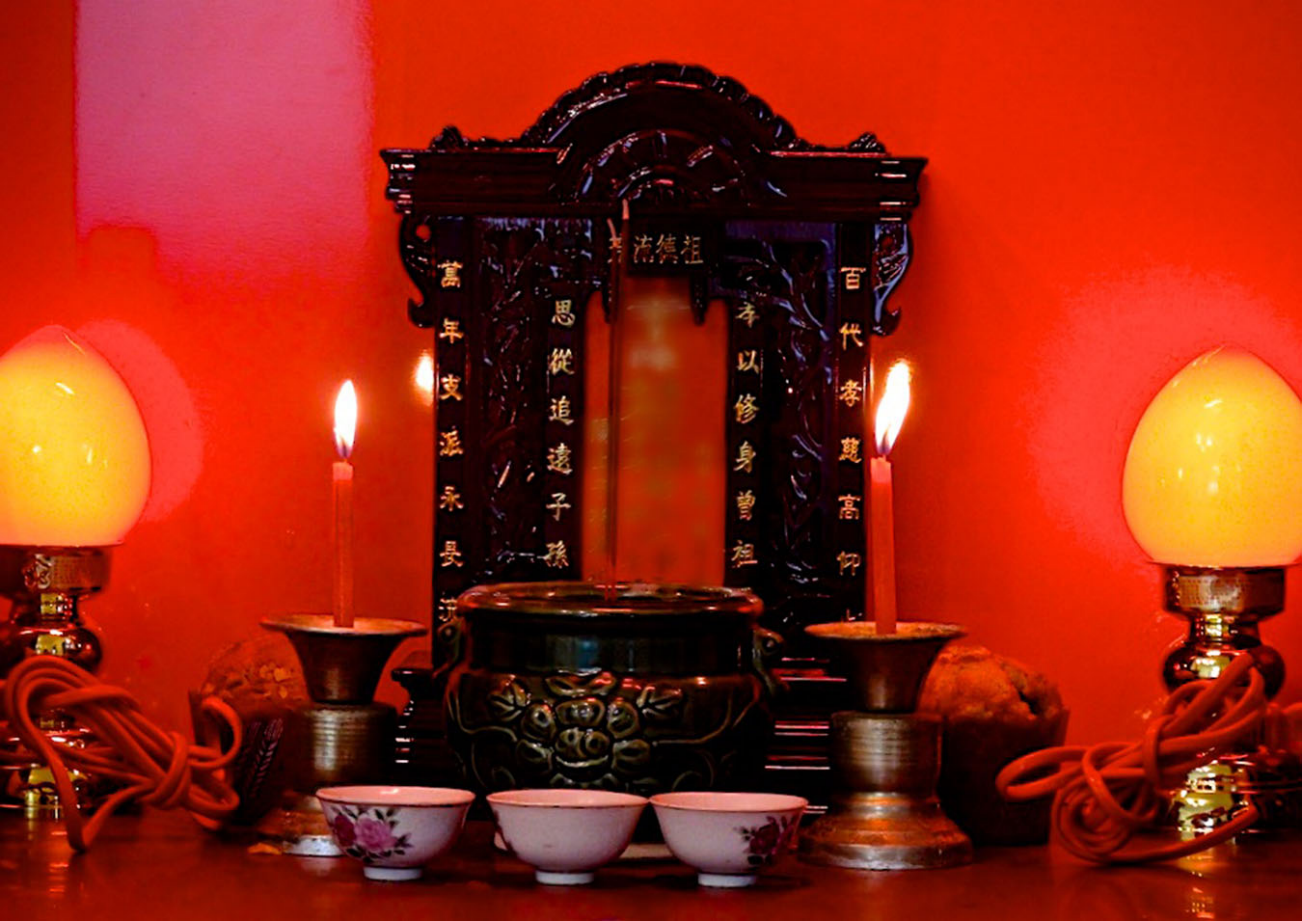
Aunt Zheng's new ancestral altar. (Photo by the author.)

Aunt Zheng paid 20,000 NT dollars – about 500 British pounds – for what she described as a simple ancestral altar, new ritual accessories such as lamps, cups, an incense vase, a wooden case, and, of course, new tablets. This amount excluded the 3,800 NT dollars (about 100 British pounds) that she handed in a red envelope to the geomancy expert, who later performed rituals to install her new altar in the new flat according to the principles of *fengshui*. ‘This is the altar I chose for my new home’, said Aunt Zheng, pointing at a small but cosy wooden ancestral table (Fig. 13), when I accompanied her to a shop specializing in ancestral tables and other ritual objects. ‘I chose a simple one’, she told me. ‘There is no space for the gods (*shen*), only space for the ancestors (*zuxian*). The geomancy master thinks what counts is to have the ancestors within one's home. I can still pray to the gods (*shen*) at the temple’.

Yet, some of the wealthiest families went big and spent a fortune for huge, finely decorated altars. In the high‐rise blocks, the size and decor of the altar marked a family's status, making higher military rankings immediately visible through the allocation of their comparatively expansive four‐room flats, sized 34 *ping*.^10^ Lower‐ranking residents followed with three‐room houses, sized 30 *ping* and 28 *ping*, respectively. The inhabitants of the large four‐room flats placed their altars in a dedicated room treated as a proper ancestral hall, while those in the smaller flats arranged altars in the living room, often suspended from the wall.

However modest Aunt Zheng's table may have been ‘without the space for the gods’, she had taken the important decision to preserve her husband's ancestral tablets, and with them his family history. Other women, though, had opted neither to move their ancestral altar to their new home, nor to buy a new one. Among them was Aunt Li, who explained that: ‘The ancestral table needs a direction (*fangxiang*)’. She told me that locating an ancestral altar in a new flat was not a simple task as she could not have just casually positioned it in the new apartment. Since the ancestral altar would have needed to face a specific cardinal direction, it was unlikely that it would have fitted in her new living room, or that it would have matched her plans for decorating it.

Worshipping in new buildings would have been even more problematic since the apartment block regulations forbade incense burning within the home, let alone the darkening of the new, white‐painted ceilings that would follow the use of incense in no time. Aunt Li donated her ancestral tablets to a Buddhist temple, where they would be taken care of. Besides the relationship each household had established with its forebears over time, different attitudes towards ancestors stemmed from a new lifestyle in the residential setting.

Aunt Jiang equally stopped the tradition, though for different reasons. Since her children had left home, she felt worshipping was too much work for herself alone. She concluded that placing the ancestral tablets in an ancestral hall (*dingwutang*), where they could be worshipped continuously, would be best for the whole family. Abandoning the practice of worshipping ancestors in her home, she left behind a repository of family history that would otherwise have been typical of Taiwanese domestic spaces. After considering the new domestic spatial arrangements – including new regulations, aesthetic canons, and habits (cf. Tamburo 2020) – Zhongxin families alternately discarded their ancestral altars or donated or relocated them elsewhere. The decision to abandon ancestral worship resulted in a rupture not only with tradition but also with the material repository of patriliny and mainlander ancestry in the domestic space.

‘Many people are not bringing their ancestral altars into their new homes; many families are placing the tablets in temples or ancestral halls’, said Uncle Wang's daughter while a geomancer was installing her new altar in the flat, adding that ‘The cult of ancestral worship does not fit the modern apartment and modern life’. However, for those families who preserved them, altars acted as objects of repair that restored the ruptured relationship between veterans and their ancestors in the new homes. For the Wang family, history remained paramount. Among its deceased, the Wang family counted Uncle Wang's wife and one of his sons. Sister Wang pointed out that their ancestral altar thus performed important repair work, reconnecting the new home to multiple generations of the household's deceased family members:

I want them to have a new place, and this is a way to show them you finally have something in your life they can be proud of. They could not see this house when they were alive, but we can live with them in the new house now. Who knows, maybe this new house will bring us more luck in life.

Rebecca Bryant observes that ‘objects have a temporal dynamism’ that ‘points to the potential, the possible, the eventual, in ways that sometimes may overlap with experiences of the past’ (2014: 684). New ancestral tables were new possessions for some Zhongxin villagers that became imbued with family history. Their altars exemplified aspirations for a modern lifestyle, the transmission of family lore, and connections to family history and ancestors across time and space. As bearers of their household's pasts and futures, these altars become important platforms through which the residents moved from clearing their homes to buying things anew, and from remembrance to aspiration.

## Conclusion

Throughout this article, I have proposed that the relocation of an urban village in Taiwan unfolded as a time of social reproduction, even in the face of impending social and cultural loss. To this end, I have thrown new ethnographic light on the importance of biographical objects among the inhabitants of Zhongxin, who set out to repair the life‐changing ruptures they had faced, first, through resettlement as military exiles in Taiwan, and, second, through their relocation to high‐rise flats sixty years later. Biographical objects such as military awards, gifted blankets, clothing, furniture, and ancestral altars (both new and old) enable residents to reconstitute their personhood vis‐à‐vis the relocation and enable Taiwanese women especially to direct acts of care and aspiration towards the social reproduction of the household.

My analysis has revealed that to gain a full understanding of the gendered labour of reproducing the family, it is necessary to marry the anthropology of kinship to the study of materiality and displacement. Here, the value, sentiments, repair work, and aspirations invested in old biographical objects like Aunt Zheng's family ancestral altar, and new ones such as furniture and ritual objects purchased for their latest home, provide the ethnographic grounding needed to expand the analytical purchase of certain foundational concepts within the anthropology of Chinese kinship and relatedness – from the ‘cycles of yang’ that underpin intergenerational transmission to the gendered labour of care in reproducing patriliny. My ethnography has shown that, especially during moments of relocation, Taiwanese women do not simply reproduce patriliny; they go a step further by transmitting the mainlander ancestries and identities of their husbands to the next generation while strengthening bonds with their children. Their intergenerational transmission of mainlander agnatic kinship shows how family memories, identities, and aspirations come to be produced within the domain of the uterine family, where reminiscing through objects is needed to repair significant ruptures to family life.

My ethnography has also shown that repair work is complex. Aunt Zheng's gifted clothing, Aunt Li's donated ancestral tablets, or even Aunt Jiang's abandoned altar and worship of her ancestors at home evidence that decisions over preserving or discarding biographical objects entail axial negotiations on the value of one's own possessions, relations, and ancestry across different gender and generations. Indeed, paying attention to the subtleties of this repair work and the claims of identity it entails is one of the very few ways left of bringing the mainlander identity in Taiwan fully into focus, as this is an identity rapidly disappearing from the island after having been routinely opposed to and separated from the wider Taiwanese identity for sixty years.

Thus, in offering an account of the sentiments, emotions, reminiscences, and memories that the inhabitants of Zhongxin invest in their biographical objects, I have shown the value of approaching the sociality of affect through repair work. For those on the verge of moving home, gendered and generational acts of care may even enable a move from remembrance to aspiration. Mainlander veterans in Taiwan may have anticipated a sense of loss and felt nostalgia for life in the village, but their Taiwanese wives directed these reminiscences so that they could unfold as acts of care and repair family ruptures. Relocating the future, then, need not exclusively be experienced as a time of loss and nostalgia, for it is also a time of aspiration and family reproduction, as Taiwanese women show through their everyday acts of care.

## NOTES

1 I use pseudonyms for all my interlocutors named in this article.

2 Dominic Yang (2020: 64) estimates the number of refugees from mainland China who reached Taiwan to have been around 1.2 million, much less than the often cited 2 million. According to Yang's historical records, among these refugees, 582,086 people belonged to the KMT military.

3 Taiwan's political landscape may be divided into two large coalitions: the conservative pan‐blue camp is headed by the KMT and advocates for Taiwan's reunification with China, while the pan‐green camp is led by the Democratic Progressive Party (DPP) – the historical opposition party to the KMT – and favours Taiwanization and Taiwanese independence.

4 The protest movement led by students and civic associations opposed the KMT's approval of the Cross‐Strait Service Trade Agreement. They occupied the legislative chambers for twenty‐three days, from 18 March to 10 April 2014.

5 Beyond financial reasons, many soldiers exiled to Taiwan married later in life because of a marriage ban imposed on army personnel by the Nationalist government (relaxed only in the late 1950s) that sought to keep soldiers readily deployable in a counterattack on the mainland.

6 Hoklo is one of the four ethnic categories recognized in Taiwan, the others being Aboriginal People (*yuanzhumin*), Mainlanders (*waishengren*), and Hakka (*kejiaren*). The term refers to the descendants of Han Chinese who settled in Taiwan before the KMT retreat to Taiwan in 1949, and whose ancestral homes are largely located in China's Fujian province.

7 The term ‘mainlander sister’ (*dalumei*) is a pejorative for ‘mainland spouses’ and denotes ‘sexual promiscuity, desire for easy wealth, and communist infiltration’ (Friedman 2015: 7).

8 Hokkien is a Southern Min language originating in Fujian province, China, where it is widely spoken. In Taiwan, a variation of Fujianese Hokkien is the native language of the Hoklo ethnic group.

9 Guanyin is one of the most well‐known Boddhisattvas in Mahayana Buddhism, Avalokiteśvara Boddhisattva, also known as the Goddess of Mercy. In Taiwan and Chinese folk religion, Tudigong is the deity protective of a locality and its respective communities.

10 A *ping* is a unit of measurement equal to about 3.3 square metres.
